# Staged Osteotome Sinus Floor Elevation for Progressive Site Development and Immediate Implant Placement in Severely Resorbed Alveolar Bone: A Case Report

**DOI:** 10.1155/2013/310931

**Published:** 2013-10-22

**Authors:** Saad Al-Almaie

**Affiliations:** KFMMC, Dental Department, P.O. Box 946, Dhahran 31932, Saudi Arabia

## Abstract

This case report discusses osteotome sinus floor elevation (OSFE) and immediate placement in 2 stages in severely resorbed alveolar bone height in which multiple implant placement is not otherwise feasible due to a lack of initial stability. The first implant placed using OSFE without bone grafting prepares the adjacent resorbed sites for further implant placement in the sinus areas, which allows for better initial stability and early functional loading. This process avoids the conventional extensive lateral approach for sinus lifting and bone grafting procedures even in extremely resorbed alveolar bone.

## 1. Introduction

Osteotome sinus floor elevation (OSFE) is a widely practiced technique for elevation of the maxillary sinus prior to implant placement [1, 2]. Summers suggested that OSFE techniques should be performed with immediate insertion of the implant when the remaining bone measures over 6 mm in height [3, 4]. Jensen recommended that the procedure should be performed with simultaneous implant placement when a residual subsinus alveolar bone height (RSBH) of at least 5 mm is present [5]. When less than 5 mm of RSBH is present, primary implant stability may be compromised [6]. The implant is inserted simultaneously with a sinus lift procedure when sufficient primary stabilization can be expected [7, 8]. 

The osteotome technique is a less invasive method of elevating the sinus floor, reduces the need for more traumatic and expensive procedures, and decreases the risk of damaging the Schneiderian membrane [9]. However, OSFE exhibits a lack of initial stability in severely resorbed alveolar bone [10]. Most cases of prosthetic rehabilitation of the severely posterior maxilla require multiple implants. Attaining initial stability in cases of severely resorbed bone is difficult and is often complicated by fractured residual bone during multiple implant insertion. The reduced initial stability and the delay in further implant placement cause considerable delays in the functional loading of the implant. We report a case of multiple implant insertion on a severely resorbed alveolar bone height in a staged manner in which the first implant is placed by tenting the sinus membrane using OSFE without a bone graft to prepare the adjacent resorbed sites for further implant placement in the sinus areas, which allows for better initial stability and early functional loading. 

## 2. Case Report

A 30-year-old male with a noncontributory medical history presented for the replacement of missing upper-left first and second premolars and molars, which were extracted 3 years earlier. The results of blood tests, including a complete blood count, chemistry, and clotting profile, were all within normal limits.

**Figure 1:**
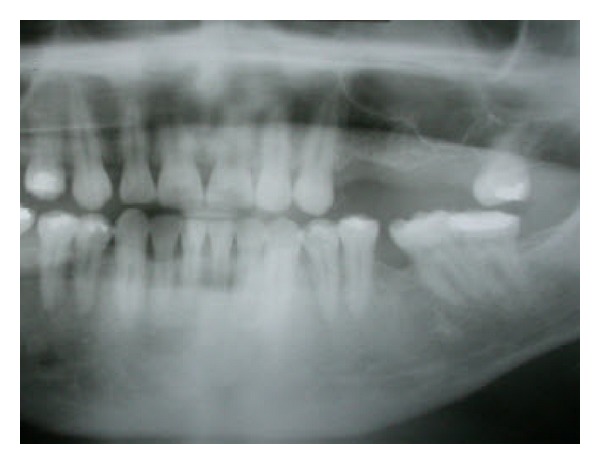
Baseline orthopantomogram showing that the subantral bone height was limited at the maxillary posterior left quadrant, with severely resorbed alveolar bone height at the site of the first maxillary molar.

The initial orthopantomogram (Figure 1) revealed an alveolar bone height resorbed edentulous area in the upper-left posterior maxillary region with pneumatization of the maxillary sinus. The residual bone height was 2.5 mm (class D) between the upper first and second molars, 4.5 mm (class C) in the second molar area, and 7 mm (class B) in the second premolar area. The classification of sinus morphology (classes A through E) was initially proposed by Jensen to aid in the selection of the appropriate grafting material or technique at a specific site [5]. The alveolar ridge was approximately 8 to 10.5 mm in width, and the occlusal clearance was 6 to 7 mm. 

In the first stage, the 1st implant was placed at the least alveolar bone height, which was located in the 1st molar area, to obtain maximum lifting of the sinus area and avoid any interference with the Schneiderian membrane in the 2nd stage and prepare the adjacent resorbed sites for further implant placement in the sinus areas. The punch incision was initially preferred (the alveolar ridge was approximately 8 to 10.5 mm in width) but because of the risk of miscalculating the exact alveolar bone height in that critical area, under local anesthesia, a full-thickness flap was elevated using a mid-crestal incision and 2 releasing buccal and palatal incisions of the upper-left first molar. The bone was accessed and marked with a round bur. A 2.2 mm diameter pilot drill was used to the required depth of 0.5 mm below the roof of the sinus. Radiographs were taken with a depth gauge and distance indicator to determine the length of the preparation.

**Figure 2:**
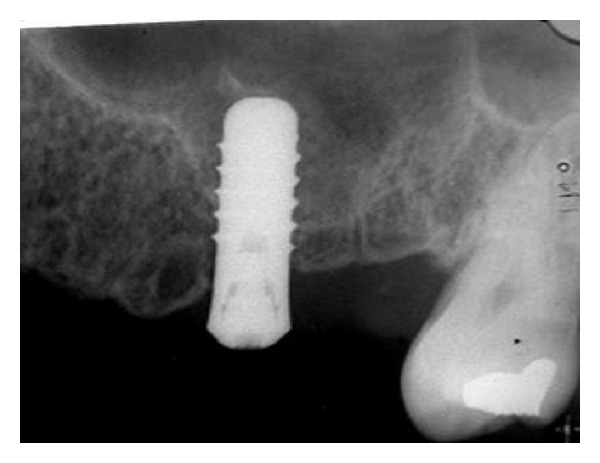
Radiograph taken after completion of the OSFE procedure without bone grafting with 8 mm lifting of the sinus membrane and a residual bone height of less than 3.0 mm and the amount of sinus floor elevation.

To improve the primary stability in cancellous bone, bone condensation through radial reinforcement was attained by a series of bone condensation devices with a tapered tip and an appropriate diameter of 2.2 mm up to 4.2 mm to widen the implant bed (ITI osteotome instruments for bone condensation, Institut Straumann, Basel, Switzerland). Malleting was performed to fracture the bottom of the sinus cavity using a 4.2 mm angled osteotome with a concave tip (ITI instrument for partial sinus floor elevation, Institut Straumann). A change in the resonance during malleting indicated complete osteotomy. The Valsalva test to assess the patency of the Schneiderian membrane was negative throughout the procedure. A 12 mm long, 4.8 mm diameter, standard neck, and nonsubmerged, SLA, screw-type ITI implant (Institut Straumann) was used. Manual and gentle screwing of the implant facilitated lifting of the sinus membrane to the required implant height, and the initial stability was attained. The surgical site was closed using 4-0 silk sutures. The implant position, osteotomized portions of the sinus floor, and the amount of sinus floor elevation were visible on radiographs (Figure 2). Amoxicillin, 500 mg per 8 h, analgesics, and chlorhexidine mouthwash twice daily for 7 days were prescribed for the patient. The sutures were removed after 10 days, and the patient was followed up once monthly for 3 months.

**Figure 3:**
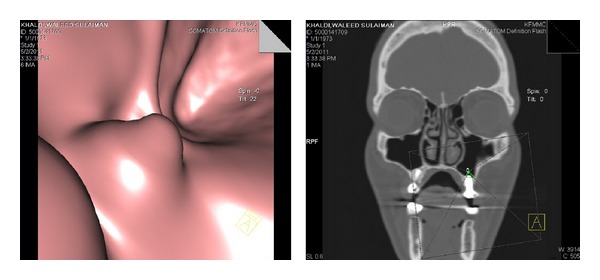
The CT and DentaScan reformatted flythrough image of the staged osteotome sinus floor elevation showing the intact Schneiderian membrane over the projection of the apical border of the first implant.

Three months after the first implant was placed in the upper-left first molar area, a reformatted flythrough image of the maxillary sinus was obtained using computed tomography (CT) and DentaScan; reformatted cross-sectional images were obtained for the radiographic evaluation of the inferior wall of the maxillary sinus and apical border for the first implant at area of the upper-left first molar after the osteotome sinus floor elevation (Figure 3) to reveal the Schneiderian membrane over the apical border projection of the first inserted implant. 

Four months after the first implant was placed, punch incisions were used to access the underlying alveolus for further implant placement. These sites required more than 5 mm of faciolingual width and little or no site development. A small envelope incision or tissue punch can be made to expose the bone for the osteotome; however, punch incision can be less traumatic and time consuming, with fewer complications and faster soft tissue healing. By including soft tissue thickness and using a surgical guide in the surgical plan, the procedure was accomplished and resulted in better patient comfort and easier surgical care. Three implants were placed on either side of the first implant. (i) A 12 mm long, 4.1 mm diameter, standard neck, nonsubmerged, SLA, screw-type ITI implant was used in the area of the upper-left second premolar. (ii) A 10 mm long, 4.1 mm diameter, standard neck, nonsubmerged, SLA, screw-type ITI implant was used in the area of the upper-left second molar. Both implants were placed using sinus floor elevation with the osteotome technique. (iii) A 12 mm long, 4.1 mm diameter, standard neck, nonsubmerged, SLA, screw-type ITI implant was placed in the upper-left first premolar area away from the maxillary sinus. 

**Figure 4:**
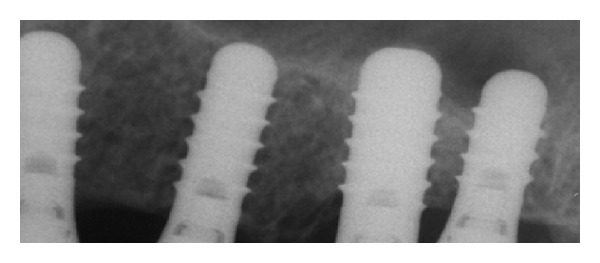
Postoperative radiograph of the left maxillary quadrant four months after the placement of three implants with better initial stability medially and distally to the first implant showing the new sinus floor with all the implants appropriately positioned and inclined.

**Figure 5:**
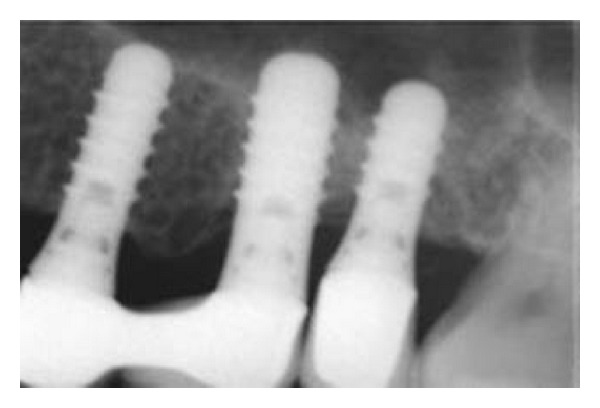
Periapical radiograph after 12 months of prosthetic loading showing a stable clinical situation in the area around the apex of the implants.

**Figure 6:**
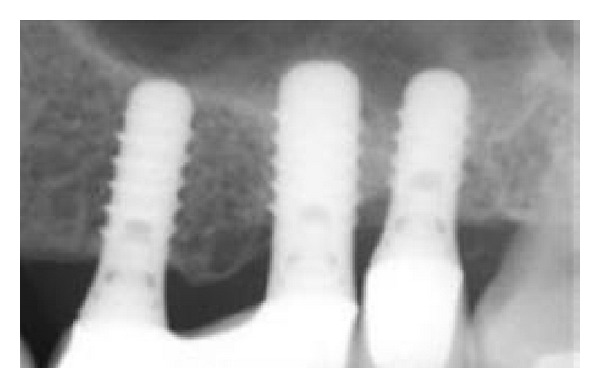
Periapical radiograph after two years of prosthetic loading showing a stable clinical situation in the area around the apex of the implants. A dome-shaped structure was apparent at the sites of the first and second molars.

**Figure 7:**
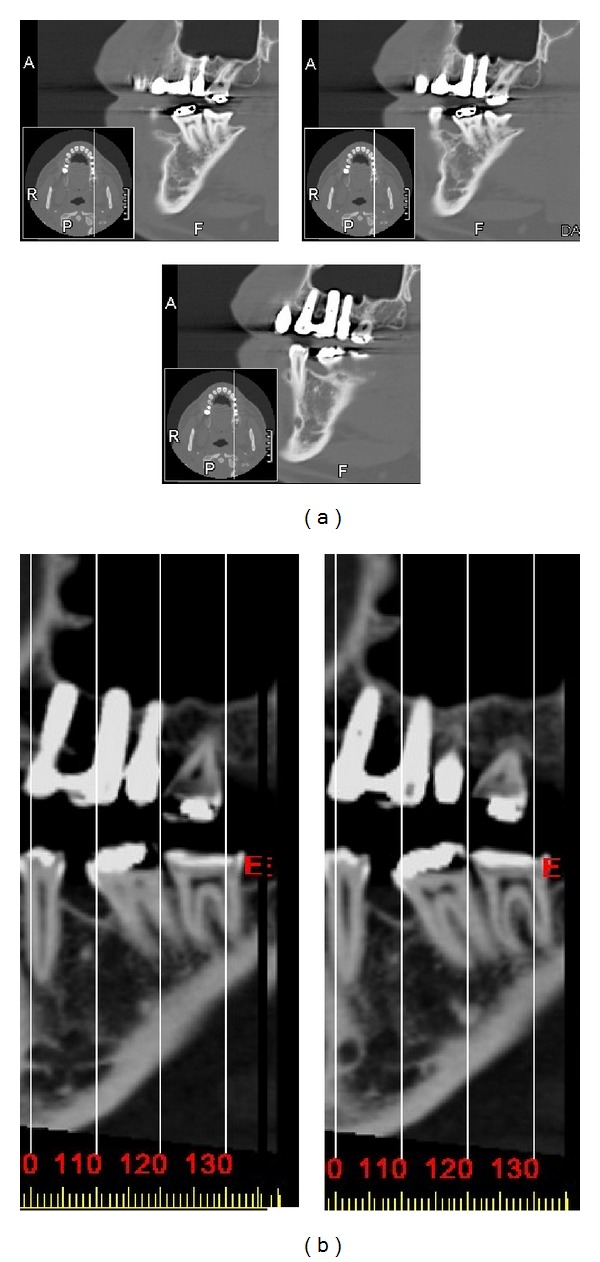
(a) CT scans showed the implant and surrounding bone and the new sinus floor using the staged osteotome sinus floor elevation technique on the left side 2 years after the operation. (b) Selected magnifying images for CT scans showed the implant, apical borders for these implants, surrounding bone, and the new sinus floor using the staged osteotome sinus floor elevation technique on the left side 2 years after the operation.

Four months after inserting the implants, radiographs were taken and they showed the implant and surrounding bone under the tented sinus membrane in the upper-left first molar region (Figure 4). The radiographs also showed the transformation of the residual ridge from classes C and D to classes A and B at the medial and distal sides of the implant, respectively. All the implants were loaded and restored with porcelain-metal retainers. Radiographs taken at 12 and 24 months (Figures 5 and 6) showed a stable clinical situation in the area around the apex of the implants. A dome-shaped structure was observed at the sites of the first and second molar areas. Different CT scans, which were obtained after 24 months postoperatively, were used to show the implant, surrounding bone, and new sinus floor using the osteotome sinus floor elevation technique (Figures 7(a) and 7(b)). 

## 3. Discussion

In patients with a severely resorbed maxilla, minimally invasive sinus floor elevation with simultaneous implant placement using osteotomes does not appear to be the method of choice. A 2-stage procedure using a lateral window technique [11, 12] or a crestal core approach [13, 14] is preferred. The OSFE procedure involves initial fixation of the implant using the residual alveolar ridge. A residual bone height of less than 4 mm is associated with reduced primary implant stability [15, 16], and a minimum of 5 mm of preoperative bone height has been suggested by many authors [17, 18]. Toffler reported that the primary factor in predicting implant survival using OSFE procedures was the residual height of the alveolar ridge and its ability to stabilize the implant [19]. 

According to the principles of guided bone regeneration, membrane elevation with space maintenance and blood clot formation may be sufficient to obtain neoformation of bone in the newly created space [20]. Lundgren et al. placed implants protruding into the sinus without introducing a grafting material and showed that all the implants achieved sufficient osseointegration and stability after 1 year of loading [21]. Nedir et al. used an expansion osteotome instead of drills to avoid ovalization of the osteotomy site and to condense the surrounding bone. Implants were often placed deeper with the flared neck resting against the crestal bone, which increased the stability [22].

In the present case report, implant installation was possible with a maxillary bone height of approximately 3 mm using relatively long implants (10 and 12 mm sink depth). Initial stability of the first implant was attained by lateral condensation. The lateral condensation technique performed using a series of osteotomes promotes better initial stability [3, 9, 20]. The quality of bone with a dense cortical plate increases the initial stability. In addition to thread engagement, the body design and surface roughness of the implants provided a frictional interface with the receptor site to assist in the mechanical retention by facilitating bone ingrowth during osseointegration [23]. In the presented case, we used the atraumatic, round-shaped end of the ITI implant, which limits the risks of damaging the sinus membrane during the sinus lift osteotome procedure. The convex apex shape of the implant is designed to prevent the tearing of the sinus membrane.

Buser et al. [24] suggested that the omission of tapping in low-density bone would improve primary implant stability. Other authors have proposed using osteotome bone condensation [25] with a smaller than recommended final drill size [26] or placing a submerged implant with its collar in a supracrestal position. Vidyasagar et al. [27] avoided tapping and cervical flaring to improve the primary stability of the implant. Avoiding cervical flaring at the preparation site for a placed dental implant even in low-density bone increases the initial implant stability. In the present case report, tapping and cervical flaring were avoided to increase the initial implant stability. Nedir et al. reported that tapered implants with a reduced thread pitch were placed with good primary stability in the atrophic maxilla of 2 patients using an osteotome sinus floor elevation procedure without grafting material [10].

Biscaro et al. observed signs of osseointegration near the apical third of the implant. A cortical wall was present at the apical end of the implant, which suggests the formation of a new sinus floor [28]. The regenerative properties of the bone beneath the sinus floor resulted in high endo-sinus bone gain. Some researchers have reported successful sinus elevation without bone grafting, and for all the studied implants, the osteotome procedure without grafting material was effective in forming new bone beyond the original limits of the sinus [10, 22, 29, 30]. In the present case report, the least alveolar bone height (less than 3 mm) for the OSFE and immediate placement for just 1 implant was selected because it is a new procedure and any failure would not jeopardize or complicate the entire quadrant if more implants were used. Nevertheless, the lateral window procedure can be used for any Schneiderian membrane perforation or complications that cannot be managed by the 1st stage OSFE procedure. Elevating the Schneiderian membrane with simultaneous implant placement is sufficient for creating bone beyond the natural limit of the sinus, and that was observed in the 3-month radiological study. This procedure created bone beyond the natural limit of the sinus and facilitated further implant placement by conventional OSFE without risking the loss of initial stability or other complications, such as fractures of the antral floor during malleting. 

For patients, there are several advantages of staged OSFE for progressive site development and immediate implant placement in severely resorbed alveolar bone compared to the lateral window procedure: less pain and discomfort, a brief recovery, and a shorter time to implant loading. From the doctor's perspective, OSFE is a shorter procedure that can be accomplished in an outpatient setting, requires less surgical skills, time, and equipment, and is free from any meaningful complications. The success of this case in the short term suggests that an atrophic maxilla could be treated with a surgical procedure other than the classic lateral window opening for sinus augmentation. After this procedure is refined, it will likely generate a higher patient and practitioner acceptance rate than the current lateral window procedure. This study does have limitations. It describes just 1 case report and describes an evolving method that will require confirmation regarding its procedural success and failure rates, the quality and quantity of the newly formed bone over time, and implant survival rates.

## 4. Conclusion

Meticulous management of the available residual bone using a staged OSFE in conjunction with immediate implant placement is introduced in this case report. Elevating the Schneiderian membrane with simultaneous implant placement is sufficient to create bone beyond the natural limit of the sinus and allows the antral floor to be amenable to the placement of more implants in the second stage. The attained initial stability favored the early functional loading of the placed implants. The conventional traumatic lateral approach for sinus lifting and grafting procedures can be avoided even in highly resorbed alveolar bone by using this technique. More patients and a longer follow-up period to evaluate the fate of the newly formed bone over time are needed to investigate the reliability of this technique.

## References

[1] Chanavaz M (1990). Maxillary sinus: anatomy, physiology, surgery, and bone grafting related to implantology—eleven years of surgical experience (1979–1990). *The Journal of Oral Implantology*.

[2] Smiler DG, Johnson PW, Lozada JL (1992). Sinus lift grafts and endosseous implants. Treatment of the atrophic posterior maxilla. *Dental Clinics of North America*.

[3] Summers RB (1994). A new concept in maxillary implant surgery: the osteotome technique. *Compendium*.

[4] Summers RB (1994). The osteotome technique: part 2—the ridge expansion osteotomy (REO) procedure. *Compendium*.

[5] Jensen OT, Jensen OT (1999). Treatment planning for sinus grafts. *The Sinus Bone Graft*.

[6] Ten Bruggenkate CM, van den Bergh JPA (1998). Maxillary sinus floor elevation: a valuable pre-prosthetic procedure. *Periodontology 2000*.

[7] Tatum OH Maxillary sinus grafting for endosseous implants.

[8] Tatum H (1986). Maxillary and sinus implant reconstructions. *Dental Clinics of North America*.

[9] Summers RB (1994). The osteotome technique: part 3—less invasive methods of elevating the sinus floor. *Compendium*.

[10] Nedir R, Nurdin N, Szmukler-Moncler S, Bischof M (2009). Osteotome sinus floor elevation technique without grafting material and immediate implant placement in atrophic posterior maxilla: report of 2 cases. *Journal of Oral and Maxillofacial Surgery*.

[11] Fugazzotto PA (1994). Maxillary sinus grafting with and without simultaneous implant placement: technical considerations and case reports. *The International Journal of Periodontics & Restorative Dentistry*.

[12] Wheeler SL, Holmes RE, Calhoun CJ (1996). Six-Year Clinical and Histologic Study of Sinus-Lift Grafts. *International Journal of Oral and Maxillofacial Implants*.

[13] Fugazzotto PA (2001). The modified trephine/osteotome sinus augmentation technique: technical considerations and discussion of indications. *Implant Dentistry*.

[14] Toffler M (2002). Staged sinus augmentation using a crestal core elevation procedure and modified osteotomes to minimize membrane perforation. *Practical Procedures & Aesthetic Dentistry*.

[15] Hirsch JM, Ericsson I (1991). Maxillary sinus augmentation using mandibular bone grafts and simultaneous installation of implants. A surgical technique. *Clinical oral Implants Research*.

[16] Bernardello F, Righi D, Cosci F, Bozzoli P, Soardi Carlo M, Spinato S (2011). Crestal sinus lift with sequential drills and simultaneous implant placement in sites with <5 mm of native bone: a multicenter retrospective study. *Implant Dentistry*.

[17] Kang T (2008). Sinus elevation using a staged osteotome technique for site development prior to implant placement in sites with less than 5 mm of native bone: a case report. *International Journal of Periodontics & Restorative Dentistry*.

[18] Greenstein G, Cavallaro J (2011). Transcrestal sinus floor elevation with osteotomes: simplified technique and management of various scenarios. *Compendium of Continuing Education in Dentistry*.

[19] Toffler M (2004). Osteotome-mediated sinus floor elevation: a clinical report. *International Journal of Oral and Maxillofacial Implants*.

[20] Winter AA, Pollack AS, Odrich RB (2003). Sinus/alveolar crest tenting (SACT): a new technique for implant placement in atrophic maxillary ridges without bone grafts or membranes. *International Journal of Periodontics & Restorative Dentistry*.

[21] Lundgren S, Andersson S, Gualini F, Sennerby L (2004). Bone reformation with sinus membrane elevation: a new surgical technique for maxillary sinus floor augmentation. *Clinical Implant Dentistry and Related Research*.

[22] Nedir R, Bischof M, Vazquez L, Szmukler-Moncler S, Bernard J-P (2006). Osteotome sinus floor elevation without grafting material: a 1-year prospective pilot study with ITI implants. *Clinical Oral Implants Research*.

[23] Buser D, Nydegger T, Oxland T (1999). Interface shear strength of titanium implants with a sandblasted and acid-etched surface: a biomechanical study in the maxilla of miniature pigs. *Journal of Biomedical Materials Research*.

[24] Buser D, Schenk RK, Steinemann S, Fiorellini JP, Fox CH, Stich H (1991). Influence of surface characteristics on bone integration of titanium implants. A histomorphometric study in miniature pigs. *Journal of Biomedical Materials Research*.

[25] Martinez H, Davarpanah M, Missika P, Celletti R, Lazzara R (2001). Optimal implant stabilization in low density bone. *Clinical Oral Implants Research*.

[26] O’Sullivan D, Sennerby L, Meredith N (2000). Measurements comparing the initial stability of five designs of dental implants: a human cadaver study. *Clinical implant dentistry and related research*.

[27] Vidyasagar L, Salms G, Apse P, Teibe U (2004). The influence of site preparation (countersinking) on initial dental implant stability, an in vitro study using resonance frequency analysis. *Stomatologija*.

[28] Biscaro L, Beccatelli A, Landi L (2012). A human histologic report of an implant placed with simultaneous sinus floor elevation without bone graft. *International Journal of Periodontics & Restorative Dentistry*.

[29] Sohn D-S, Moon J-W, Moon K-N, Cho S-C, Kang P-S (2010). New bone formation in the maxillary sinus using only absorbable gelatin sponge. *Journal of Oral and Maxillofacial Surgery*.

[30] Ferrigno N, Laureti M, Fanali S (2006). Dental implants placement in conjunction with osteotome sinus floor elevation: a 12-year life-table analysis from a prospective study on 588 ITI implants. *Clinical Oral Implants Research*.

